# The Value of Abdominal Palpation in Labour.—I

**Published:** 1909-04-17

**Authors:** 


### Obstetrics.

### THE VALUE OF ABDOMINAL PALPATION IN LABOUR.—I.

Much has been written in the last decade of the great value of the external abdominal method of investigation in the diagnosis of presentations in labour, and its real value is appreciated by all who practise it. It is true, however, that outside of the lying-in hospitals and charities it is not used to any great extent, even by men who have been carefully taught to practise it in their student days. The reason for this is clear: abdominal palpation is difficult at first, and unless every opportunity of practising it is embraced, the difficulties are not overcome, and its true value and importance are not grasped. It may be that some patients object to it because they do not expect to be examined in that manner. Such objections, however, can be easily overcome by any man who starts by obtaining the complete confidence of his patient.

Unfortunately the habit of attempting to diagnose presentations by vaginal examinations alone not only is fallacious and leads to absolutely erroneous diagnoses sometimes, but is dangerous to the patient on other grounds. The more vaginal examinations during labour, the greater is the risk of introducing septic material from without; and in a case where there is any difficulty in making out the presentation many vaginal examinations will probably be made as long as any doubt remains. There can be no reasonable doubt that the comparatively high mortality and higher still morbidity in lying-in women in private practice compared with that in hospital practice is, at least in part, due to the number of vaginal examinations made during labour. Further, and not less important, the vaginal method leads not infrequently to erroneous diagnoses, and consequently to difficulties in treatment which might well have been foreseen and avoided. It cannot be said that abdominal examination will obviate all difficulties in diagnosis or prevent all puerperal sepsis; but it will do away with many of the former and prevent much of the latter. It can be said with absolute certainty that the position of the child and the exact presentation can be made out by abdominal palpation alone in practically all cases except those in which the face and brow present. This must not be taken to mean that a face presentation cannot be made out from above; it very often can, but not always.

The method of carrying out abdominal palpation is simple enough if two chief points are remembered—namely, that it is required to locate the head of the fœtus and to find to which side its back points. First, to locate the head: stand by the side of the patient, who, of course, lies on her back; place the hands on either side of the uterus, with the fingers pointing towards the pubes; then clip the fingers behind the symphysis, and in a vertex presentation the hard rounded head will be felt. In a primipara the head feels as if it filled the pelvis, and is usually quite low down, the greatest bulk of it being already through the brim. In a multipara the head is higher up, and may not have engaged at all. Finding that the head presents *and fills the pelvis*, a vertex presentation is certain, and it only requires now to find out which. The position of the back and limbs will be the guide to this. Starting with the hands as placed originally, they should be drawn upwards so as to grasp the greatest bulk of the uterus between them, and then by pressing alternately one side and the other the great resistance of the back will be felt, or the small parts (*i.e.* legs and arms) will become apparent. In anterior positions of the occiput the back is easy to locate, but in posterior positions it may not be felt at all, in which case the anterior shoulder and side of the fœtus must be made out. One shoulder will always be more easy to feel than the other; in fact, the posterior one can never be felt abdominally. Of the posterior positions of the vertex the right is said to occur twenty times to once of the left in every hundred vertex cases. After this, then, is it necessary to make a vaginal examination at all? Certainly not for presentation diagnostic purposes; but in the first stage it may be necessary to gauge the dilatation of the cervix and the integrity of the membranes. Unless, then, there is some other urgent reason, one, or at the most two, vaginal examinations should be the limit in an uncomplicated vertex case.

When, on making the first abdominal palpation, the vertex is not found in the lower uterine segment, the next movement must be to find out if it is near the fundus or lying near the iliac fossa. To find the head at the fundus the finger-tips of the examiner should be turned towards the fundus and the hands spread out at the sides of the uterus as before. The head at the fundus is usually to one side or the other of it, not exactly in the middle. It is easily recognised from its hardness, its shape, and its mobility, the latter perhaps being the great diagnostic point between the head and the breech. The breech cannot be moved or "ballotted" on the trunk, the head can. Finding this, the position must be pelvic, and it makes very little difference whether the actual presentation is a complete breech, footling, or knee. If the head cannot be found at the fundus, then it must lie nearer the side of the uterus, and will usually be found pointing towards one or other iliac fossa.

As far as errors of diagnosis go, perhaps the greatest is to mistake a breech for a face presentation, and yet this has occurred sufficiently often when vaginal examinations only are made. Such a mistake ought to be impossible if the abdominal method is practised. The commonest mistake is to overlook an occipito-posterior position of the vertex, and this mistake is *the* one of all others which leads to prolonged labour, severe maternal injuries, and is perhaps the commonest cause of intrapartum death of the infant. An occipito-posterior position cannot be overlooked if abdominal palpation is properly carried out. Two conditions make abdominal palpation difficult, and may make it impossible—namely, great obesity and hydramnios.

